# The Early Diagnosis of Pulmonary Tuberculosis

**Published:** 1901-07-20

**Authors:** 


### THE EARLY DIAGNOSIS OF PULMONARY TUBERCULOSIS.

The striking results which have followed the careful employment of sanatorium treatment even in cases of acute and distinctly ulcerative phthisis show that one must not despair of arrest even where the disease is comparatively advanced. Still, taking the disease as a whole one cannot but see that the true *rôle* of the sanatorium and its methods is as a means of cure in incipient tuberculosis rather than as a means of producing arrest or mere relief in advanced consumption. So far then as therapeutics aim at cure the interest more than ever centres in early diagnosis. We must not, however, make light of the difficulties which exist in discovering tuberculosis at the stage at which it is most amenable to treatment. Often in the early stages of the disease the symptoms are not such as to point to the lung at all. Loss of appetite and loss of weight and strength may be for long the only symptoms of the invasion of tubercle, and unfortunately the frequency with which these symptoms are accompanied by an obvious anæmia or a troublesome dyspepsia, by which they may seem to be explained, is very apt to lead the investigator astray in his diagnosis. Dr. J. J. Perkins,[1] writing on this subject, says, "In taking the history of their cases from any large number of phthisical patients one cannot but be struck with the frequency with which they describe a stage of vague ill-health as the commencement of their illness, and the precursor by many weeks or even some months, of truly respiratory symptoms such as cough. The frequency of this absence or late appearance of cough and expectoration at the onset of phthisis is not at all sufficiently realised." Far too often such cases are lightly dismissed or reassured. Again, so impressed is the practitioner apt to be with the essentially chronic nature of phthisis as he usually sees it, that its very suddenness of onset in some cases is apt to divert suspicion. What, then, are the signs that one must look for in the earlier stages of tuberculosis, at a time when the condition is one of invasion of the lung, starting in the wall of the bronchus from which small areas of consolidation or tuberculous nodules have started, but in which no extensive consolidation or softening has occurred. First there are the ordinary physical signs of very slight consolidation. To these, however, must be added two conditions which should always awaken suspicions—tachycardia and hyperæsthesia. The heart rate is often, if not usually, raised to 100 or 120 in early tuberculous infiltration of the lung. This is not due to the presence of fever, nor is it the result of increased work, necessitated by destruction of the lung, for it occurs in the very earliest stages of the disease. Hyperæsthesia over the affected apex is often observed on percussion, even the slightest. "There is a temptation," says Dr. Perkins, "to look upon this with contempt as a neurotic manifestation, but in reality it is highly significant, and is a referred pain from the damaged and irritated lung below . . . its presence should always lead to a very careful examination of the corresponding apex." The signs may be slight, but then the condition which they indicate is as yet still slight. But their cumulative weight when all pointing in the same direction is great, and when they occur in a person out of health they are quite sufficient to demand further investigation; especially is this the case in regard to evening fever, loss of weight, and night sweats. No great rise of temperature is to be looked for, a persistent evening temperature of 99·5° or 100·5° F. being quite sufficient to indicate disease. The temperature should be taken between 4 P.M. and 6 P.M., and in cases of doubt every four hours. If we could always find tubercule bacilli it might be thought that the answer to all our doubts might be at once obtained by examining the sputum. In most cases it is so, and this is a mode of inquiry which ought to be employed as a routine in all cases of persistent cough; but experience shows that pulmonary tuberculosis may advance to a considerable degree, even so as to give well-marked physical signs, and yet be without expectoration. Thus there are cases in which some other decisive test of the presence of tubercle is wanted, and for this purpose the use of tuberculin has come into renewed favour of recent years. The *modus operandi*, says Dr. Perkins, is as follows:—The temperature of the patient is taken frequently for several days or a week, and averaged. A dose of 0·5 or 1 milligramme of the old tuberculin is then injected. If the result is positive, a reaction, *i.e.*, some malaise and a rise of temperature up to 2° F. follows. If the result is negative, after waiting two or three days, twice the original dose is injected; and if this again is negative, after waiting three more days, an injection of 5 milligrammes is given. In most cases the reaction, if resulting at all, follows the smaller injection. The rise of temperature usually subsides in twenty-four hours. There seems to be a general consensus among the leading authorities of all countries as to the absence of danger in this proceeding. The method has not yet come into much favour in this country, but we cannot shut our eyes to the praise accorded it on the Continent and in America. It is to be hoped that the approaching Congress will give some pronouncement as to its safety; and also as to its infallibility, for the reaction is said by some to occur in the syphilitic which rather complicates matters. Two further signs of pulmonary tuberculosis are mentioned by Dr. Perkins which require the most careful consideration, namely pleurisy and hæmoptysis. By hæmoptysis he means the initial hæmoptysis, on which phthisis so often follows, occurring in people apparently in the best of health.

The absence of physical signs, which is usual in such cases, and the fact that after the accident is recovered from the patient often regains for a time his usual health, lead to the condition being seriously underestimated by many men. "The chest is examined, nothing is found, and hence nothing is considered to be present. Really one should not expect to find any physical signs, and should not be misled by their absence. Too often, however, as the result of this negative examination phthisis is excluded and the patient reassured, and allowed to pass out of sight without a word of warning." Such patients should be treated as though they already had demonstrable phthisis. With the knowledge we now have, what used to be called idiopathic pleurisy (not empyema but pleurisy with effusion occurring independently), must be taken as a sign of tuberculosis. The same is probably true of dry pleurisy. Such patients then should be treated as if they were tuberculous; in fact, pleurisy may well be regarded as one of the physical signs of tuberculosis. With this reservation, however, that it does not of necessity mean that the disease has so far attacked the lung, the primary seat of the tubercle from which the pleura has become invaded being the bronchial glands. If we do not go so far as to regard all cases of pleurisy as tuberculous we must trust to the use of tuberculin to aid in differentiating them. In days of old a doctor did best to keep his suspicions to himself in cases of early phthisis. If he laid down a proper *régime* and gave some tonic and cod-liver oil he had done what he could, and the worst thing that he could do was to depress the patient by the use of the dread word consumption. But now that we have some hope of curing the disease if it can but be caught early enough, the position of affairs is altered. Everything depends on ensuring that the malady shall be treated seriously at the earliest possible stage, and so soon as (weighing the evidence carefully, but not waiting for absolute certainty, a thing so seldom met with in medical problems) we can satisfy ourselves that the case is one of tuberculosis, it is our duty to take care that its full gravity if left to its course, as well as its full hopefulness if at once treated, shall be made plain to those who are responsible.

[1] Practitioner, July.

